# Editorial

**Published:** 1867-12

**Authors:** 


### Editorial.

CLOSE OF THE VOLUME.—The present number closes the Eighth Volume of the EXAMINER. Severe sickness in our little family circle, prevents us from giving our intended clinical report and some editorial articles. We can, at present, only thank our patrons for their past favors, and express the hope that they may be continued.

TRANSACTIONS OF THE ILLINOIS STATE MEDICAL SOCIETY.—The Transactions of our State Medical Society for 1867, are now ready for distribution to the members. The publication has been delayed, at least two months, by the late reception of the report on Plastic Surgery.

PROFESSIONAL APPOINTMENTS.—The following changes have been made in the Faculty of the Long Island College Hospital: Dr. C. L. FORD has accepted the Chair of Anatomy, and Dr. FOSTER SWIFT, that of Obstetrics and Diseases of Women and Children. Dr. SAM'L G. ARMOR has been transferred to the Chair of Principles and Practice of Medicine. Dr. AUSTIN FLINT retaining the Chair of Clinical Medicine.

NOTICE.—*Prof. Davis:*—I am pained to announce the death of JAS. F. SPAIN, M.D., in Urbana, Ohio, on the morning of October 13th last. Dr. SPAIN was a man of superior mental and social qualities, and was known only to be loved. Dr. SPAIN graduated in Rush Medical College, in the spring of

1860, with honor to the school and himself. He died from an attack of apoplexy of the brain.

D. B. WREN, M.D.

---

### REPORT ON THE SANITARY CONDITION AND PREVALENCE OF DISEASE IN CHICAGO DURING THE MONTHS OF JULY, AUGUST, AND SEPTEMBER, 1867.

---

By N. S. DAVIS, M.D., Member of the Sanitary Committee.

---

Read to the Chicago Medical Society.

In my previous report to this Society, ending June 30th, it was stated that the meteorological and sanitary conditions during the preceding three months, had been such as to favor a good condition of public health, and a low ratio of mortality.* The last week in June was represented as hot, oppressive, with south winds during each morning, but cool and bracing with west and north-west winds in the evenings.

The first day of July was clear, cool, and bracing, with a prevalence of north-west winds. The 2d, was ushered in with a hot, oppressive atmosphere, and south wind, which was continued until 4 o'clock P.M., when it suddenly changed to the north-east, became boisterous, with clouds and thunder, but no rain. The 3d, was warm and cloudy, with slight rain in the morning, followed by west wind and copious showers of rain at evening. The morning of the 4th was hot, damp, and oppressive, with south wind. Early in the afternoon the wind changed to the north, the atmosphere became cooler, and the night was cool and clear. The 5th and 6th were moderately warm, sultry, with south and south-west wind, and a drizzling rain nearly all of both days. The 7th, was clear, dry, and cool, with northerly winds all day. The 8th, atmosphere clear, warm, and oppressive, with south wind during the morning. In the afternoon, rain fell, during which the wind changed to the north, bringing a cold, clear atmosphere in the evening; and which was con-

* See Med. Examiner, for August, 1867.

tinued through the 9th. The morning of the 10th was ushered in with a strong south wind, hot and oppressive, with showers of rain at midnight. The 11th, was cloudy, damp, moderately warm, with wind, first south-west and then west. The 12th and 13th, mostly clear, dry, and cool, with light winds from the west and north. The 14th, was clear, hot, and oppressive, with south wind all day; but during the night, the wind changed to the north-west, bringing a cold, drizzling rain, which lasted until noon of the 15th, when the atmosphere became cool, clear, and dry, and continued so until the morning of the 17th. From the 17th to the 22d, the atmosphere was mostly clear, moderately hot, with cool nights, and very little wind from any quarter. There were clouds and slight rain on the 19th. The night of the 22d, like the day, was hot, clear, oppressive, with only slight wind from the south. These atmospheric conditions continued until the evening of the 24th, when there was slight rain, followed by thunder and slight rain on the morning of the 25th, with a continuance of high heat and dampness of atmosphere. In the evening, the wind changed to the north-west, bringing first light showers, and afterwards a cool, clear atmosphere, which continued until afternoon of the 26th, when the wind changed to the south, and the heat and dampness again became very oppressive, and continued so until the morning of the 28th. From this date until the evening of the 30th, a strong west and north-west wind prevailed, with a cool, dry, and bracing atmosphere. During the night of the 30th, the wind changed to the south, bringing a copious warm shower at 4 o'clock A.M. of the 31st. The heat and dampness continued until 3 o'clock P.M., when the wind suddenly changed to the north-west, accompanied by an active thunder shower, and followed by a temperature so low as to be very chilly.

It is thus seen, that the prominent meteorological characteristics of July were, frequent changes in temperature, in atmospheric currents, and frequent light falls of rain, with a mean average temperature for the month about ordinary, it being several degrees lower than the mean temperature of July 1866; and slightly above that of the same month in 1865.

In regard to local sanitary conditions, it may be said, that much greater efforts had been made to keep the streets and alleys free from garbage and decomposable materials, and to prevent the out-door privies from becoming full, than in previous years. Yet these efforts on the part of the Board of Health were only partially successful, there being during all the month, in some populous portions of the city, unopened ditches, full privies, and other materials capable of decomposition under a July temperature. The gross mortality for the month was 538, being 168 less than for the same month in 1866, and 113 more than in July 1865. The increase over 1865, is mostly owing to the increase of population, while the excess in 1866 was the direct result of a prevailing epidemic influence, of a choleraic character. This is shown by the following comparison:

| | Whole number of deaths from bowel-affections in | | |
|---|---|---|---|
| | 1865 | 1866 | 1867 |
| July, | 163 | 299 | 236 |

More than 80 per cent of this large mortality from bowel-affections in July of each year, occurred in children under three years of age. The connection of this excessive infantile mortality with the first high temperature of every summer, is shown by comparing the mortality in July in each year with that of June. Thus:

| | The mortality from bowel-affections in | | |
|---|---|---|---|
| | 1865 | 1866 | 1867 |
| June, | 20 | 45 | 21 |
| July, | 163 | 299 | 236 |

A more detailed observation of facts, further shows that a large proportion of the attacks in children commence on particular days, when the temperature of both day and night is high, with an excess of atmospheric moisture. Thus, of 73 attacks of cholera-infantum, diarrhœa, etc., coming under my own observation, during July, 1867, 56 were found to have commenced on the following days, namely, 18 on the 3d and 4th; 10 on the 10th and 11th; 11 on the 13th and 14th; 7 on the 19th; and 10 on the 22d and 23d. By referring to the meteorological facts already given, it will be found that these are the days preëminently characterized by high temperature, oppressive south winds, and excessive moisture.

The first case of well-marked, spasmodic cholera that came under my observation during the month, was a little girl, in the rear of 351 Illinois Street, who was attacked during the hot, sultry morning of the 4th, passed rapidly into collapse without medical treatment, and died on the morning of the 6th. The next was a man, in the rear of 124 South Jefferson Street, who was attacked with serous diarrhœa on the 11th, which culminated in the full choleraic symptoms during the night of the 13th. Two cases occurred on the 20th: one was a woman in the rear of 331 Fourth Avenue; the other was a child, 2 years of age, in the rear of 24 West Harrison Street. Both these patients passed rapidly into collapse and died. The fifth case commenced on the 22d, in an intemperate man, living at 119 South Market Street. On the 23d, two cases occurred: one, a middle-aged woman, at 197 Van Buren Street; the other, also a female, about 30 years of age, residing at 70 West Indiana Street. Both these were violent cases, and had passed into collapse before I saw them. They died on the morning of the 25th. Another well-marked case occurred, in a woman in advanced life, at 202 Sebor Street, on the night of the 24th. She was placed under treatment early, and recovered. During the same day, a man, at 342 West Kinzie Street, was attacked with cholera diarrhœa, which ended in the development of full cholera symptoms, on the 25th; but he recovered. At 3 o clock A.M. of the 31st, a man was attacked with active cholera symptoms, at 54 Fourth Avenue, but he recovered. The cases here alluded to as cholera, were as well characterized in their symptoms as any cases that occurred during the summer and autumn of 1866, and they were, consequently, reported to the Board of Health.

During the last week of the month, I saw many cases of dysentery, in which the evacuations were bloody serum instead of mucus, accompanied by cool extremities, small pulse, and rapid prostration, constituting what has been called cholera dysentery.

In making inquiries and noting carefully, from day to day, the exact commencement of cases of cholera-infantum, cholera-morbus, and spasmodic cholera, not only during the month now under consideration, but during the summer months of every year since 1849, I have fully satisfied myself that fifteen out of every twenty cases of these diseases have their first symptoms developed during days and nights when the atmosphere is hot, damp, and deficient in free electricity; or during the night time, in persons while sleeping in small, close, unventilated bedrooms. It is well known to every observing practitioner, who has seen much of cholera-infantum and epidemic cholera, that a large proportion of the attacks commence between midnight and 6 o'clock A.M. And I have gathered sufficient data to show that the impure air of small or overcrowded and unventilated sleeping rooms, is one of the most potent exciting causes determining such results.

*August.*—From the 1st of August to 11 o'clock A.M. of the 4th, the atmosphere was cool, dry, and nearly free from clouds, with a predominance of west and north-west winds. At 11 o'clock of the 4th, the atmosphere became still, or moved only by a slight breeze from the south, and hot and oppressive. It continued in the same condition until the afternoon of the 9th, when, suddenly, a brisk, cool wind came from the north, with flying clouds and slight rain. The 10th and 11th were cool and dry, with north winds. With the morning of the 12th came south wind and a hot, dry atmosphere, followed by copious showers and sharp lightning in the evening. From the 13th to the 16th, the air was cool, dry, and pleasant, except about four hours of hot, oppressive atmosphere on the afternoon of the 14th. The 17th and 18th, were continuously hot and oppressive, with very slight wind from the south. The morning of the 19th was ushered in by a copious warm shower, speedily followed by a cool east, and, later, north-east wind, with a cool, rainy afternoon. From the 20th to the 25th, inclusive, the atmosphere was cool and bracing, except two or three hours in the middle of each day, which were hot and oppressive. The wind veered each day from south-east in the morning, to north-west and north in the evening. Moderate showers fell in the evenings of the 21st and 22d, accompanied by fine displays of atmospheric electricity. With the morning of the 26th, came a warm shower, with south wind: hot and oppressive during the middle of the day, followed by copious showers and sharp lightning in the evening. From the 27th to the 30th, inclusive, the atmosphere was cool, dry, and pleasant, with chilly nights. The 31st was cold and rainy, with south-west wind.

It is thus seen that the prominent meteorological characteristics of August were, a predominanee of northerly winds, cool, bracing air, and a full average of atmospheric electricity. There were only eleven hot, oppressive days during the month, and only four of these were consecutive or continuous, one with another. Rain fell on seven different days, accompanied by thunder and lightning on four.

During the first six days of the month, I met with no cases of either cholera-morbus or cholera-infantum, except such as had dated their commencement in July. But the continuous high temperature from the 4th to the 8th was accompanied by quite a number of new attacks on the 7th and 8th. During the warm days and cool nights from the 9th to the 16th, attacks of typhoid fever and dysentery became very frequent, especially among the laboring classes. Some new cases of cholera-infantum and diarrhæa occurred from the 17th to the 19th, and many old cases that had partially recovered were renewed. From this time to the end of the month, most of the new attacks of bowel-affections, both in children and adults, presented the form of dysentery; while typhoid fever continued to be of frequent occurrence.

The only cases of cholera that came under my observation during the month, were the following:—A servant girl was attacked violently, during the hot, sultry night of the 4th, while sleeping in an overcrowded, unventilated room, in the alley in the rear of the Opera House. She was placed under treatment early and recovered. A young man at 44 North Peoria Street, who had just returned from the country, was attacked during the latter part of the night of the 17th. He was placed under treatment almost immediately and recovered. The same morning (18th) I was called to an Irish laborer, at No. 48 Ohio Street, whom I found in a state of complete collapse, and he died during the same day. He had some diarrhœa since the evening of the 14th, and all the symptoms of violent epidemic cholera supervened on the morning of the 17th. On the morning of the 19th, saw another Irish laborer in the same neighborhood, 77 Ontario Street, who commenced having diarrhæa at noon on the 18th, which developed into full and severe cholera during the latter part of the following night. Although passing to the very verge of collapse, he recovered. The local sanitary conditions in the neighborhood where these two cases occurred were bad. The drainage was very imperfect, several of the out-door privies were full to the surface, and a cow was stabled under the house No. 48 Ohio Street. On the 29th, I was called to see a man, at 645 State Street, who had been attacked with diarrhœa at Racine, about ten days previous. He was brought to the city on the 27th, when his symptoms assumed the character of severe spasmodic cholera. On the 29th, I found him with entire suppression of urine, pulseless, cold, very blue and corrugated on the surface, and all the phenomena of complete collapse. He died soon afterwards. It will be observed that all the foregoing cases, except the one brought from Racine, occurred on days or nights when the atmosphere was hot and oppressive, with south winds.

The gross mortality for the month of August was 697, of which 340 were from bowel-affections, namely, cholera-infantum, 212; cholera-morbus, 7; cholera, 2; diarrhœa, 85; dysentery, 36. From personal observation, I am certain that a large majority of the fatal cases of cholera-infantum were such as had their origin in July and had lingered in a chronic form until some time in August. And, on a strict diagnosis, a large proportion of them should have been classed as dysentery. Compared with the same month for 1865 and 1866, the result is as follows:

| 1865. | | 1866. | | | 1867. | |
|---|---|---|---|---|---|---|
| Gross Mort. | Bow.-Aff. | Gr. Mort. | Bow.-Aff. | Cholera, | Gr. Mort, | Bow.-Aff. |
| 464 | 208 | 940 | 376 | 139 | 697 | 340 |

We thus see, that while July and August of 1867 give a mortality much below the same months of 1866, it is so much above that of 1865 as to indicate a higher ratio in proportion to the population.

We have occupied so much time and space with the meteorological and other details concerning the months of July and August, that we will make only a brief allusion to September. It was characterized by no striking meteorological characteristics. The atmosphere was, a great part of the time, dry, warm in the middle of the day and cool during the night. The rainy days were few, and the amount of rain-fall below the average for this month in this locality. Bowel-affections in young children rapidly diminished, while dysentery and continued fevers increased among adults. This is shown by the following comparison:

| | Cholera-Infantum. | Dysentery. | Typhoid. | Typhus. |
|---|---|---|---|---|
| July, | 177 | 10 | 9 | 2 |
| August, | 212 | 37 | 13 | 5 |
| September, | 82 | 29 | 18 | 4 |

The total mortality for September was 507; being 232 less than in the same month of 1866, and 161 more than in September 1865. I saw, during the month, only three well-marked cases of spasmodic cholera, one of which passed into fatal collapse in a few hours. The effect of local causes on the general mortality is strikingly illustrated, by comparing different sections of the city. For instance, the total population of the 1st, 2d, 3d, 9th, and 10th Wards in 1866, was 63,747. The total deaths in the same Wards in August and September, 1867, was 190, or 1 to 335 of the population. The total population of 5th, 6th, 7th, 8th, 13th, and 14th Wards, was 69,670 The total deaths in the same Wards during August and September, 1867, was 555, or one to 125 of the population. The first series of Wards embraces the best drained and most substantially built portion of the city, inhabited chiefly by the mercantile, professional, and business classes of the city; while the second series is the reverse, both in sewerage and building, and is inhabited chiefly by laboring classes of Irish and German nativity.

The facts briefly, and perhaps imperfectly, set forth in this report, clearly establish two conclusions, of great etiological as well as sanitary importance:—The first is, that certain atmospheric or meteorological conditions are essential to the production of some of the most important endemic as well as epidemic forms of disease. The second is, that certain local sanitary conditions, relative to sewerage, house ventilation, privy accommodations, cleanliness, and personal habits, are capable of increasing, in a three-fold degree, the injurious and fatal effects of the meteorological conditions alluded to.

---

We cordially commend the following enterprise to the patronage of our subscribers and readers.—[Ed.

THE HALF-YEARLY COMPENDIUM OF MEDICAL SCIENCE: Being A Synopsis of Practical Medicine, Surgery, and Medical Literature.

The first part of this work will be issued from the office of the *Medical and Surgical Reporter* on the first of January, 1868. It will comprise about 300 pages royal octavo size, and will contain a well-prepared synopsis of the articles in the medical periodicals and monographs, and a general review of the medical literature of the preceding six months, both of this country and Europe.

In the preparation of this work, we will be aided by many well-known writers, among whom are Drs. L. Elsburg, Samuel R. Percy, R. E. Van Gieson, F. D. Weisse, C. F. J. Lehlbach, S. W. Gross, George H. Napheys, J. E. Garretson, W. M. Turner, A. Paul Turner, and others.

The work will be systematically arranged under the following heads—subject to such modifications as time and experience may suggest—and supplied with a *Copious Index* of subjects and authors.

I. ANATOMY AND PHYSIOLOGY.

1. Comparative Anatomy and Zoölogy; 2. General and Special Human Anatomy; 3. General and Special Physiology; 4. Embryology; 5. Pathological Anatomy and General Pathology.

II. MEDICAL PHYSICS, BOTANY, CHEMISTRY, AND TOXICOLOGY.

III. MATERIA MEDICA AND THERAPEUTICS.

1. Pharmacology; 2. General and Special Therapeutics; *a.* Electrotherapy; *b.* Hydrotherapy; *c.* Anæsthesia, etc.

IV. GENERAL MEDICINE.

1. History of Medicine; 2. Statistical Medicine; 3. State Medicine—including Forensic Medicine, Hygiene Dietetics, and Preventive Medicine; 4. Epidemiology; 5. Animal and Vegetable Parasites; 6. Medical Bibliography.

V. CLINICAL MEDICINE.

1. General and Constitutional Diseases; 2. Diseases of the Brain and Nervous System; 3. Blood Diseases; 4. Local Diseases, *a.* Respiratory Organs, *b.* Circulatory System, *c.* Organs of Deglutition and Digestion, *d.* Urinary and Male Genital Organs, *e.* Exanthematous Diseases.

VI. OBSTETRICS AND DISEASES OF WOMEN AND CHILDREN.

VII. SURGERY.

1. General Surgery—Tumors, etc.; 2. Mechanical Surgery; 3. Orthopædics; 4. Military Surgery—Wounds, etc.; 5. Fractures and Dislocations; 6. Amputations and Resections; 7. Topographical Surgery, *a.* Head, Neck, and Breast, *b.* Eye and Ear, *c.* Nose, Mouth and Throat, *d.* Abdomen, *e.* Genital Organs; 8. Diseases of the Skin; 9. Gonorrhœa and Syphilis.

VIII. VETERINARY MEDICINE.

*Terms of Subscription.*—Subscriptions will now be received at the following rates:

COMPENDIUM, per annum, two numbers,............$3 00
" single number,........................ 2 00
" and *Med. & Surg. Reporter*, per an.,- 7 00

S. W. BUTLER, M.D.

D. G. BRINTON, M.D. } Editors.

*115 South Seventh St., Philadelphia.*

We earnestly hope that this NATIONAL UNDERTAKING will be heartily supported by the profession. All who are willing to subscribe are requested to fill the following blank, and return to this office, *before the 10th of December*, as the size of the edition of the first number will be governed by the encouragement we receive.

---

MONEY RECEIPTS TILL NOVEMBER 25th, 1867.—Drs. J. Good, Warren, Ind., $3; R. M. Buchner, Aroma, Ill., 3; T. A. Bunnell, Chicago, 3,75; O. T. Maxon, Chicago, 6; John McHugh, Urbana, Ill., 2; Geo. W. Keener, Venice, Ill., 3; John Bell, Benton Harbor, Mich., 3; R. S. Lewis, Dubuque, Iowa, 3; B. Ragon, Roseville, Ill., 3; C. M. Spalding, Byron, Ill., 3; T. H. Bras, New Boston, Ill., 7; W. Stewart, Otumna, Iowa, 9.

Mortality Report for the Month of October:—

The monthly report is as follows:—

CAUSES OF DEATH.

| | | | |
|---|---|---|---|
| Accidents, | 13 | Fever, Scarlet, | 3 |
| Angina Membanacea, | 2 | Fever, Typhoid, | 25 |
| Anæmia, | 1 | Fever, Typhus, | 2 |
| Apoplexy, | 2 | Gastritis, | 1 |
| Asthma, | 1 | Hæmatemesis, | 1 |
| Aseitics, | 1 | Hepatitis, | 1 |
| Atrophy, | 2 | Heart Disease, | 4 |
| Birth, premature | 9 | Hydrocephalus, | 4 |
| " still | 26 | Inanition, | 6 |
| " tedious | 1 | Jaundice, | 2 |
| Brain Congestion of, | 5 | Laryngitis, | 1 |
| " Dropsy, | 1 | Liver, abscess of | 1 |
| " Inflammation of, | 8 | Lungs, congestion of | 1 |
| " Softening of, | 2 | Measles, | 4 |
| Bright's Disease, | 1 | Meningitis, | 5 |
| Bronchitis, | 2 | Meningitis, Cerebro Spinal, | 3 |
| Bowels, inflammation of | 5 | Meningitis, Tubercular | 2 |
| Bowels, obstruction of | 1 | Mortification, | 1 |
| Croup, | 11 | Nephrites, | 1 |
| Cerebritis, | 1 | Old Age, | 4 |
| Chills, congestive | 1 | Opthalmia purulent, | 1 |
| Convulsions, | 42 | Paralysis, | 2 |
| Cholera-Morbus, | 2 | Consumption, | 25 |
| Cholera Infantine, | 41 | Peritonitis, | 4 |
| Cyomosis, | 1 | Pneumonia, | 11 |
| Debility, | 7 | Poison, | 1 |
| Delirium Tremens, | 1 | Phelebitis, | 1 |
| Diarrhœa, | 11 | Scrofula, | 2 |
| Diphtheria, | 10 | Small-Pox, | 20 |
| Dropsy, | 1 | Suicide, | 1 |
| Dysentery, | 13 | Stomach, cancer of | 4 |
| Enterites, | 8 | Tabes Mesenterica, | 13 |
| Epilepsy, | 2 | Teething, | 14 |
| Eucephalis, | 1 | Whooping-Cough, | 2 |
| Fever, Bilious | 1 | Ulcers, malignant | 1 |
| Fever, Malignant | 1 | Ulcers, cancer of | 3 |
| Fever, Termittent, | 1 | | |
| Fever, Puerperal, | 5 | Total, | 428 |

COMPARISON.

| | |
|---|---|
| Deaths in October, 1867, | 428 |
| Deaths in October, 1866, | 1,170 |
| Decrease, | 742 |
| Deaths in September, 1867, | 507 |
| Decrease, | 79 |

Ages of the Deceased.—Under 5 years, 326; over 5 and under 10 years, 17; over 10 and under 20, 19; over 20 and under 30, 34; over 30 and under 40, 31; over 40 and under 50, 22; over 50 and under 60, 17; over 60 and under 70, 7: over 70 and under 80, 3; over 80 and under 90, 5; still born and premature, 27; tedious births, 1; unknown, 9. Total, 428.

SEXES.

| | | |
|---|---|---|
| Males, 230 | Females, 198 | Total, 428 |

COLOR.

| | | |
|---|---|---|
| Colored, 8 | White, 420 | Total, 428 |

NATIVITIES.

| | | | | | |
|---|---|---|---|---|---|
| Chicago, | 224 | England, | 6 | Scotland, | 2 |
| Other parts U. S., | 65 | France, | 1 | Sweden, | 8 |
| Austria, | 1 | Germany, | 42 | Switzerland, | 2 |
| Belgium, | 1 | Holland, | 7 | Wales, | 2 |
| Bohemia, | 4 | Ireland, | 40 | Unknown, | 1 |
| Canada, | 8 | Norway, | 7 | | |
| Denmark, | 1 | Prussia, | 6 | Total, | 428 |

---

CHESTNUT LEAVES IN PERTUSSIS.—In the *Cincinnati Lancet and Observer*, Dr. J. S. Unzicker, of Cincinnati, reports the use of a decoction of leaves of the chestnut, *Castanea Visca*, in hooping-cough. He says:

I have given it a fair trial in about thirty cases, and feel satisfied in saying that at last a remedy is found to cope with this disease. In all of these cases it gave decided relief the first two weeks. The cough is cut short, and patients rest easier through nights, and the decline of all symptoms from that time on is very rapid. My method of using it is as follows: take from ℥iij. to ℥iv. of the leaves to the pint of water; let it come to a boil, then pour the whole into a teapot, without straining, and let them drink occasionally—either cold or warm—and as much as they will through the day and at bedtime. Childern, I find, like to drink it, even without sugar, which I consider best, and have that way administered it to infants, without the least difficulty.

---

### WINTER RETREAT FOR CONSUMPTIVES.

A large house is being fitted up under the superintendence of DR. PRINCE, in Jacksonville, heated by furnaces, so arranged as to admit of the purification and medication of the air of the house, producing an artificial climate, not only favorable for the most speedy recovery of patients undergoing surgical treatment, but also adapted to secure the best atmosphere for those affected or threatened with pulmonary complaints.

It is expected that the repairs and alterations necessary for this purpose will be completed by the first of December.

Address DAVID PRINCE, M.D., dec1t. *Jacksonville, Ill.*

